# Pan-cancer analyses reveal the genetic and pharmacogenomic landscape of transient receptor potential channels

**DOI:** 10.1038/s41525-022-00304-1

**Published:** 2022-05-25

**Authors:** Tao Pan, Yueying Gao, Gang Xu, Ping Zhou, Si Li, Jing Guo, Haozhe Zou, Qi Xu, Xiaoyan Huang, Juan Xu, Yongsheng Li

**Affiliations:** 1 College of Biomedical Information and Engineering, Hainan Women and Children’s Medical Center, Hainan Medical University, Haikou, 571199 China; 2 Department of Radiotherapy, the First Affiliated Hospital of Hainan Medical University, Haikou, 571199 China; 3 College of Bioinformatics Science and Technology, Harbin Medical University, Harbin, Heilongjiang 150081 China

**Keywords:** High-throughput screening, Genomic engineering, Cancer epigenetics

## Abstract

Transient-receptor potential (TRP) channels comprise a diverse family of ion channels, which play important roles in regulation of intracellular calcium. Emerging evidence has revealed the critical roles of TRP channels in tumor development and progression. However, we still lack knowledge about the genetic and pharmacogenomics landscape of TRP genes across cancer types. Here, we comprehensively characterized the genetic and transcriptome alterations of TRP genes across >10,000 patients of 33 cancer types. We revealed prevalent somatic mutations and copy number variation in TRP genes. In particular, mutations located in transmembrane regions of TRP genes were likely to be deleterious mutations (*p*-values < 0.001). Genetic alterations were correlated with transcriptome dysregulation of TRP genes, and we found that TRPM2, TRPM8, and TPRA1 showed extent dysregulation in cancer. Patients with TRP gene alterations were with significantly higher hypoxia scores, tumor mutation burdens, tumor stages and grades, and poor survival. The alterations of TRP genes were significantly associated with the activity of cancer-related pathways. Moreover, we found that the expression of TRP genes were potentially useful for development of targeted therapies. Our study provided the landscape of genomic and transcriptomic alterations of TPRs across 33 cancer types, which is a comprehensive resource for guiding both mechanistic and therapeutic analyses of the roles of TRP genes in cancer. Identifying the TRP genes with extensive genetic alterations will directly contribute to cancer therapy in the context of predictive, preventive, and personalized medicine.

## Introduction

Transient-receptor potential (TRP) channels comprise a diverse family of ion channels, which play important roles in regulation of intracellular calcium. Calcium signaling pathway plays diverse roles in cellular physiology, including cell motility, cell cycle control, autophagy, and apoptosis. Notably, perturbation of intracellular calcium signaling is involved in cancer progression and metastasis.

TRP channels have been implicated in numerous human complex diseases, including various types of cancer^1^. For example, it has been demonstrated that increased expression of TRPM7 is associated with poor prognosis and metastasis of nasopharyngeal cancer. Silencing of TRPM7 gene can also decrease the migration and invasion of metastatic cancer cells in breast cancer^2,3^. TRP channels have been found to be very promising players in prostate cancer, because their expression and activity can regulate the development and progression of cancer^1^. In the last decade, dysregulation of TRP channels has been found to play important roles in regulating cellular proliferation, differentiation, and impaired death, which resulting in expansion and invasion of cancer^4,5^. Moreover, TRP channels have been found to play important roles in various late stages of tumor progression^3^.

In addition, most TRP channels have been found to be located at the cell surface, which makes them generally accessible as potential drug targets^6,7^. Drug discovery efforts by targeting TRP channel genes or proteins have been observed in number of diseases, such as chronic cough, asthma and cancer. For example, TRP channels are attractive targets for treatment of respiratory diseases^8^. High-grade astrocytoma shows increased TRPV1 expression and high-dose capsaicin administration can kill neurons owing to the Ca^2+^ overload^9,10^. Thus, TRPV1 agonists may help to eradicate brain tumor. Moreover, oesophageal and head-and-neck cancer patients also overexpress TRPV1 and TRPA1 and these cancers are more promising targets for TRPV1 or TRPA1 agonist therapy^11^. However, the functional roles of TRP channels across cancer types appear to be unclear. Some other large-scale pan-cancer analyses for specific gene lists have been performed, such as clock genes^12^, hippo pathway^13^, and heat shock proteins (HSPs)^14^. A comprehensive understanding of the genetic alterations and expression perturbations of TRP channels underlying cancer cell heterogeneity is necessary to elucidate cancer therapeutic targets.

Thus, we systematically characterize the molecular alterations and clinical relevance of TRP channels across 33 cancer types. We found the prevalent somatic mutations and copy number variation of TRP channels in cancer. In particular, we revealed that the deleterious mutations are likely to be located in the transmembrane regions of TRP genes. Genetic alterations are correlated with transcriptome dysregulation and associated with cancer-related pathways. Finally, we analyzed the correlation between drug activities and TRP gene expressions, providing candidate drug targets for cancer therapy. Taken together, our study provided a valuable resource that will guide both mechanistic and therapeutic analyses of the role of TRP channels in cancer.

## Results

### Global genetic alteration of TRP genes across cancer types

**Fig. 1:**
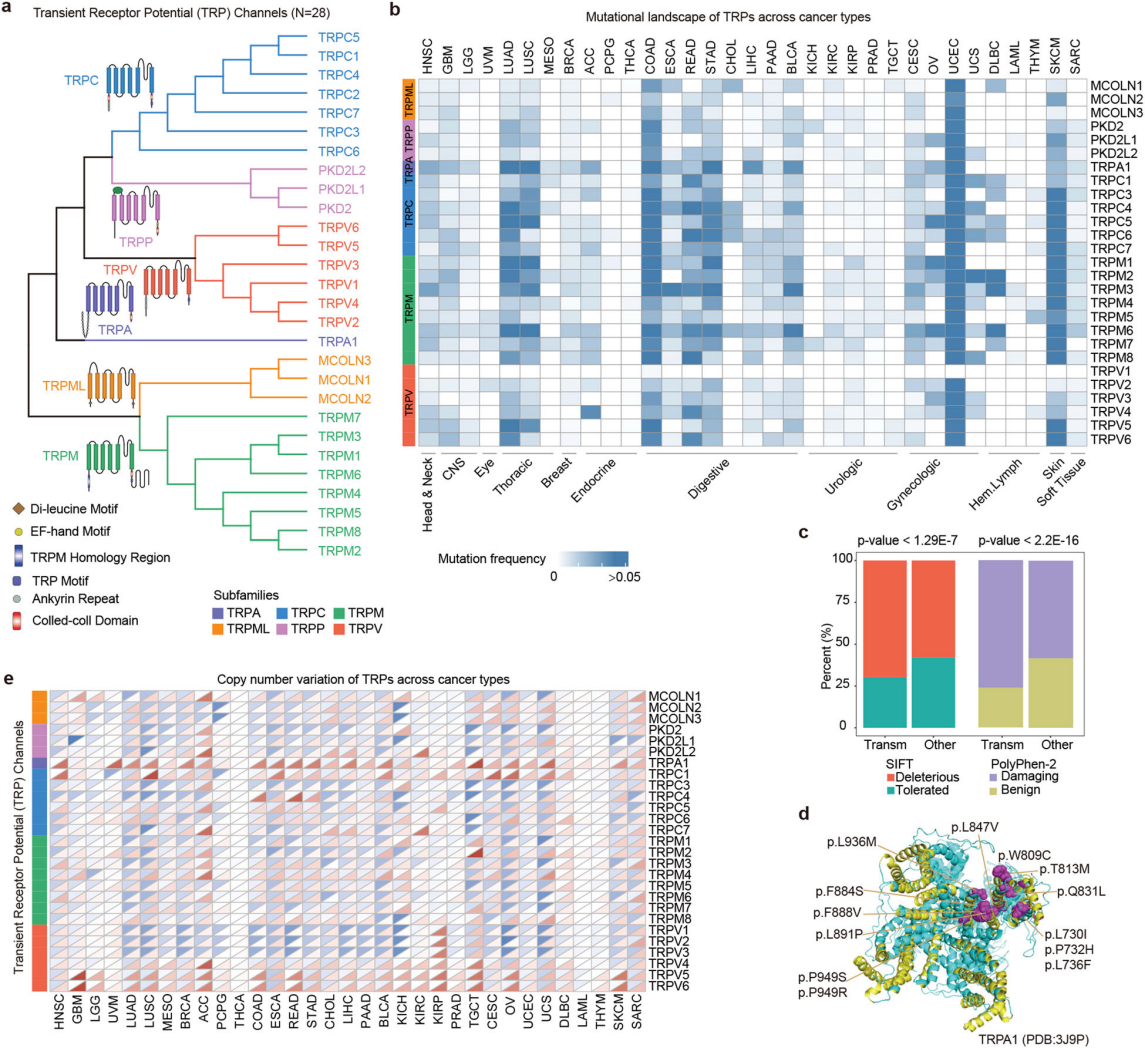
Prevalent genetic alterations of TRP genes across cancer types. **a** The evolutionary tree of TRP genes based on sequence similarity. Each branching group is representative of a subfamily of TRPs. **b** The mutation frequency profile of TRPs across cancer types. **c** Bar plots showing the proportion of deleterious or damaging mutations in transmembrane regions or other regions. *P*-values for Fisher’s exact tests. **d** PDB structure for TRPA1 and deleterious mutations were indicated with pink balls. **e** Heat map showing the frequency of CNV alterations for TRPs across cancer types. The upper of each rectangle represents the frequency of CNV loss and the bottom shows the frequency for CNV gain.

We collected 28 TRP channels genes, which were grouped into six families/sub-families (Fig. 1a and Supplementary Table 1). Sequences analysis revealed that the genes in the same family were with higher similarity. To comprehensively characterize the impact of genetic variants on TRP channels expression across different cancer types, we analyzed the somatic mutations and copy number variations (CNVs) of >10,000 patients across 33 cancer types from The Cancer Genome Atlas (TCGA). We found that the mutation frequency is relatively low for TRP genes in urological cancers, but with higher mutation frequency in thoracic, digestive, gynecologic, and skin cancers (Fig. 1b). In particular, we found that TPRA1 exhibited the highest mutation frequency (6%) across cancer types (Supplementary Fig. 1a). Moreover, we found that the results were similar to the International Cancer Genome Consortium (ICGC) data (Supplementary Fig. 2).

TRP genes are structurally similar having six transmembrane helices. We next investigated the mutation density in transmembrane regions vs. others. Although the mutation densities of transmembrane regions were similar to other regions in TRP genes (Supplementary Fig. 1b), we found that mutations in transmembrane regions were more likely to be deleterious or damaging mutations in cancer (Fig. 1c and Supplementary Table 2). For example, we identified 13 deleterious mutations in TRPA1 (Fig. 1d) and 12 deleterious mutations in TRPM2 (Supplementary Fig. 1c). We also calculated the CADD scores for mutations and found that mutations in transmembrane regions were with significantly higher CADD scores (Supplementary Fig. 3). These results suggested cancer cells might selectively alter the transmembrane regions to perturb the TRP signaling. We next investigated the CNVs of TRP genes across cancer types and found that the CNV frequencies of TRP genes are relatively higher across cancer types (Fig. 1e). For example, TRPA1, TRPC1, TRPV4, TRPV5, and TRPV6 exhibited higher CNV amplification, while TRPV1, TRPV2, TRPV3, and TRPC3 exhibited frequent CNV loss in cancer (Fig. 1e). Together, all these observations suggest that the prevalent genetic alterations of TPR genes in cancer.

### Transcriptome perturbations of TRP genes across cancer types

**Fig. 2:**
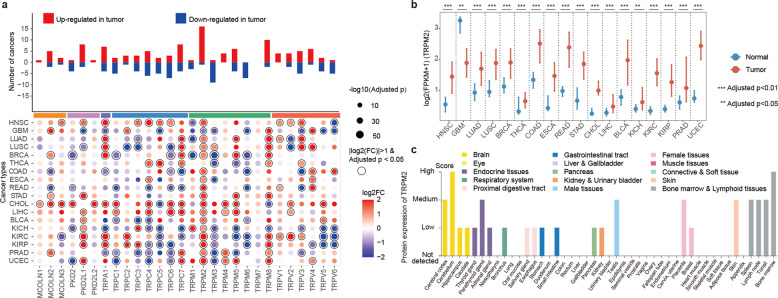
Transcriptome dysregulation of TPR genes in cancer. **a** Bar plots showing the number of cancer types that each TRP gene exhibited upregulation or downregulation. The heat map at the bottom showing the fold-changes of TPRs in comparison between cancer and normal. **b** Box plots showing the expression of TRPM2 in cancer and normal samples across cancer types. Centre line is the median, bounds of box are the upper and lower quartile. ***adjusted *p* < 0.01, **adjusted *p* < 0.05. **c** Bar plots showing the expression of TRPM2 protein across normal tissues. Tissues were colored by the organs.

Genetic alterations play important roles in regulating gene expression. We thus examined the expression of TRP genes across 18 cancers with more than five normal samples. Overall, 27 (96.4%) of the TRP genes are differentially expressed in at least one cancer type (Fig. 2a). Several TRP genes were consistently upregulated or downregulated in multiple cancer types. For example, we found that TPRM2 had relatively higher expression in tumor samples (Fig. 2b) and exhibited widely expression across normal tissues (Fig. 2c and Supplementary Fig. 4). TRPM2 channel protein expression level has been found to be increased in esophageal squamous cell carcinoma tumor tissue compared with adjacent normal tissue^15^. TRPM2 participated in the ROS hydrogen peroxide-induced increase in intracellular calcium, which inhibited cell proliferation and enhanced apoptosis. Moreover, we analyzed the gene expression of TRPs in 11 independent cancer types. We found that all TRP genes exhibited differential expression in at least one cancer type (Supplementary Fig. 5). In particular, TRPM2 exhibited upregulation in six cancer types (Supplementary Fig. 6). These observations suggested TRPM2 was an oncogene in cancer.

**Fig. 3:**
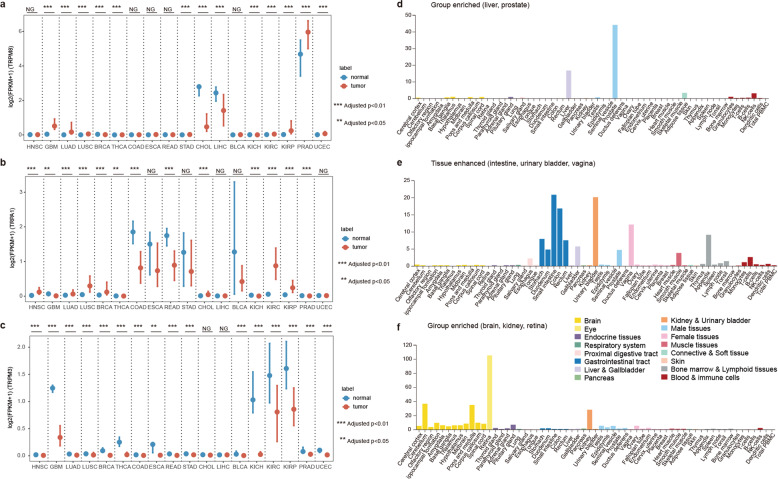
Expression of TRP genes across tissues and cancer types. **a** Expression of TRPM8 across cancers. **b** Expression of TRPA1 across cancers. **c** Expression of TPRM3 across cancers. **d** Expression of TRPM8 across normal tissues. **e** Expression of TRPA1 across normal tissues. **f** Expression of TRPM3 across normal tissues. Data of normal tissues were downloaded from human protein atlas. Tissues were colored by the organs. Centre line of boxplot is the median, and bounds of box are the upper and lower quartile. ***adjusted *p* < 0.01, **adjusted *p* < 0.05.

Moreover, we found group-enriched (liver and prostate) TRP gene TRPM8 also exhibited higher expression in liver and prostate cancer (Fig. 3a, d). Dysfunctional TRPM8 signaling has been demonstrated in the vascular response to environmental cold in ageing^16^. One recent study reinforced the importance of TRPM8 as prostate biomarker and emphasized the value of the TRP channel as promising molecular target for the treatment of prostate adenocarcinoma^17^. The tissue enhanced TRPA1 (intestine, urinary bladder, and vagina) and group-enriched TRPM3 (brain, kidney, and retina) also exhibited perturbed expression in corresponding cancer types (Fig. 3b, c and e, f). One recent study reported the endogenous expression of TRPA1 channels in human pancreatic adenocarcinoma cell lines and siRNA-induced downregulation of TRPA1 can enhance cell migration and change the cell cycle progression^18^. Moreover, emerging evidences have revealed that noncoding RNA can promote angiogenesis and metastasis through epithelial–mesenchymal transition and upregulate TRPM3 in renal cell carcinoma^19^. These observations were consistent with the recent results that tissues enriched genes or noncoding RNAs were likely to be involved in cancer^20,21^.

### Clinical relevance of TRP gene dysregulation

**Fig. 4:**
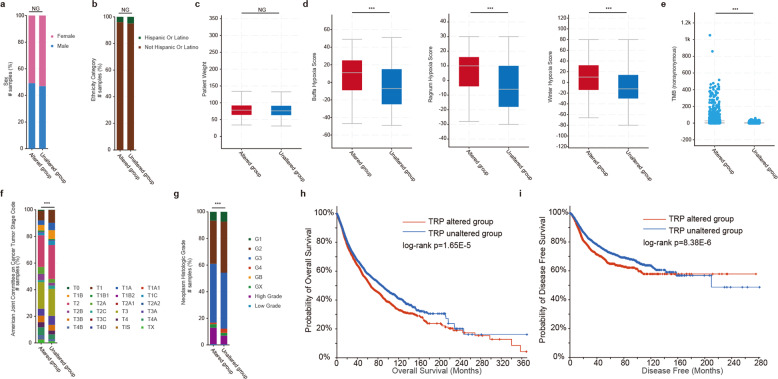
Clinical relevance of TRPs alterations across cancer types. **a** Proportions of patients with different sex in TRP altered or unaltered groups. **b** Proportions of patients with different ethnicity category in TRP altered or unaltered groups. **c** Patient weights in TRP altered or unaltered groups. **d** Hypoxia scores of patients in TRP altered or unaltered groups. **e** Box plots showing the TMBs of patients in TRP altered or unaltered groups. **f** Stages of patients in TRP altered or unaltered groups. **g** Grades of patients in TRP altered or unaltered groups. **h** Kaplan–Meier survival plot of patients grouped by with or without TRP genetic alterations. **h** for overall survival and **i** for disease-free survival. Centre line of boxplot is the median, and bounds of box are the upper and lower quartile. ***adjusted *p* < 0.01, **adjusted *p* < 0.05.

Given the global genetic alterations of TRP genes, TRP genes could provide insights into translational medicine. Although there were no significant differences in sex, ethnicity category, and weight between patients with or without TRP genetic alterations (Fig. 4a–c), we found that patients with TRP alterations had relative higher hypoxia scores and tumor mutation burden (Fig. 4d, e, *p*-values <0.001). Tumor hypoxia has been found to be a molecular hallmark across cancer types^22,23^. Moreover, patients with TRP alterations were with higher tumor stage and grade (Fig. 3f, g, *p*-values < 0.001). We found that the patients with genetic alterations of TRP genes exhibited poor survival in cancer (Fig. 4h, i and Supplementary Fig. 7, log-rank *p*-values < 0.001).

**Fig. 5:**
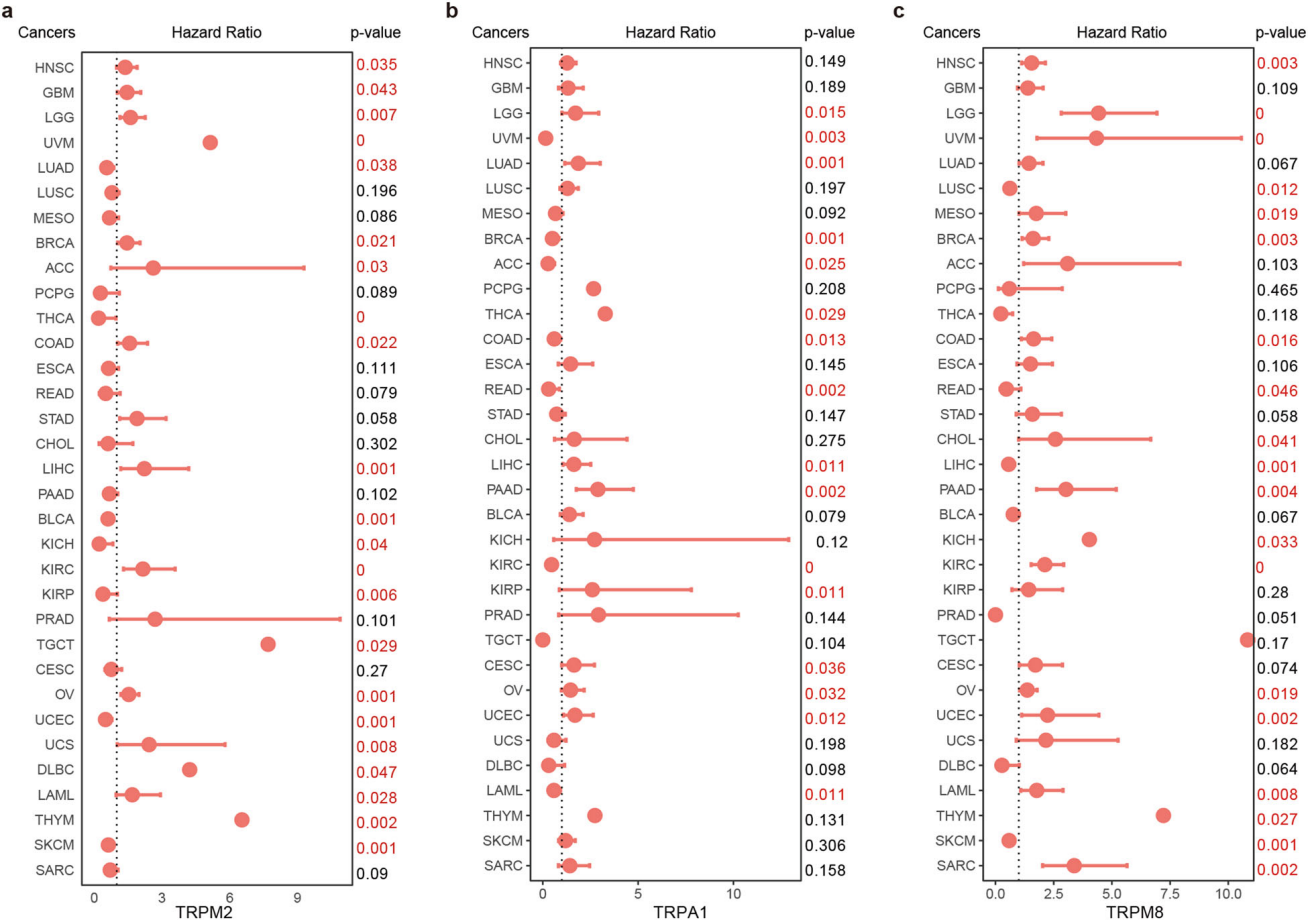
The distribution of hazard ratios across different cancer types. **a** The distribution of hazard ratios based on TRPM2 expression across different cancer types. **b** The distribution of hazard ratios based on TRPA1 expression across different cancer types. **c** The distribution of hazard ratios based on TRPM8 expression across different cancer types. Centre line of boxplot is the HR, and bounds of box are the 95% confidence levels.

Furthermore, the differential expression of individual TRP gene was associated with the clinically relevant events across cancer types. We found that the expressions of all TRP genes were associated with overall patient survival in at least one cancer type (Supplementary Fig. 8 and Supplementary Table 3). For example, the expressions of TRPM2, TRPA1, and TRPM8 were associated with patient survival in 22, 16, and 19 cancer types, respectively (Fig. 5). Moreover, we also validated the associations in independent cancer datasets (Supplementary Fig. 9). TRPM2 has been demonstrated to be participated in the ROS hydrogen peroxide-induced increase in intracellular calcium^15^. It has been shown that inhibiting the TRPA1 ion channels are critical for breaking down the oxidative stress defense system and overcome cellular resistance^24^. These results suggested potential roles of TRP genes as prognostic markers for particular cancer types.

### Functional pathways of TRP genes in cancer

**Fig. 6:**
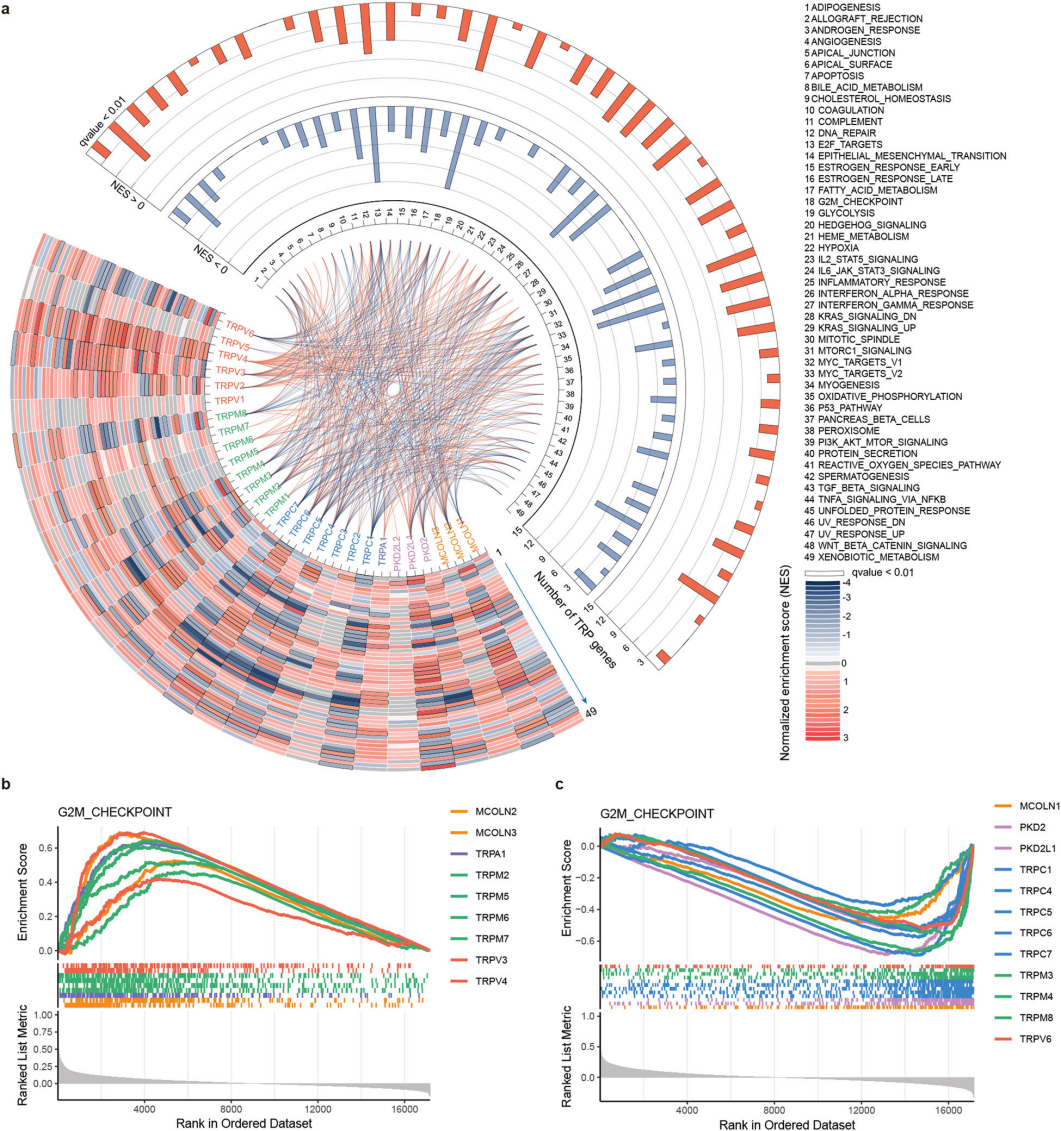
Functional pathways of TRP genes across cancer types. **a** TRP gene expression correlated with cancer pathway activities. The heat map showing the normalized enrichment scores of GSEA analysis. Pathways were indicated by numbers 1 to 49. The bar plots showing the number of TRP genes that correlated with each cancer pathway. Blue for negatively correlated and red for positively correlated pairs. The inner lines showing the significant TRP gene-pathway pairs. **b**, **c** The enrichment score (ES) distribution for the genes positively or negatively co-expressed with TRP genes in G2M checkpoint pathway. Each line is for one TRP gene and lines are colored by TRP family.

Dysregulation of expression of TRP genes has been involved in number of cancer pathways^6^. To characterize the functional pathways of TRP genes, we calculated the expression correlation of protein-coding genes with TRP gene. Gene set enrichment analysis (GSEA) was performed to find the association of TRP gene with cancer-related pathways. We found that 27 TRP genes were correlated with at least one cancer hallmark pathways (Fig. 6a and Supplementary Table 4). Moreover, we found that several TRP genes can co-regulate cancer-related pathways and the TRP genes were also significantly co-expressed and interacted with each other in protein–protein interaction network (Supplementary Fig. 10).

**Fig. 7:**
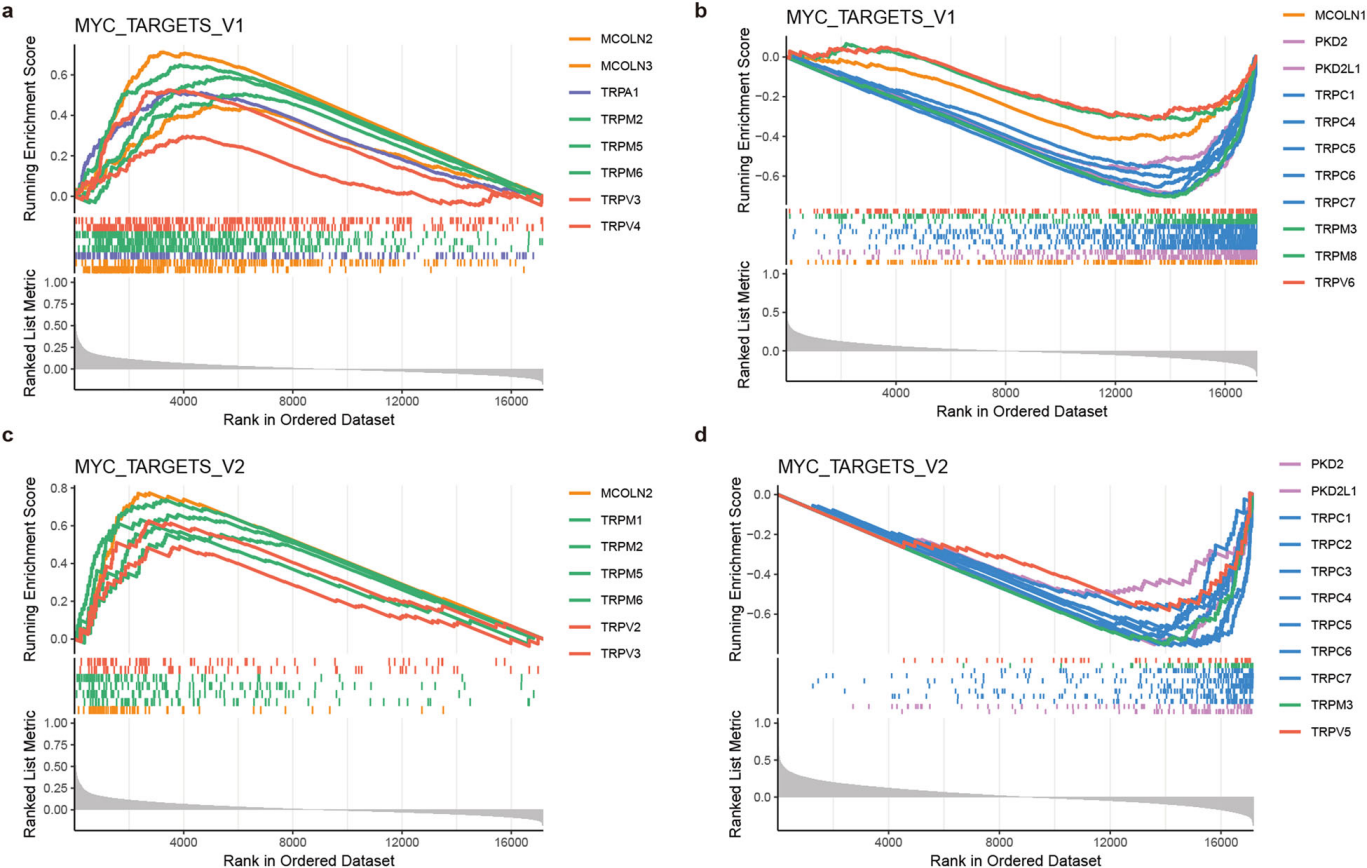
Genes co-expressed with TRPs are enriched in MYC targets. **a** The enrichment score (ES) distribution for the genes positively co-expressed with TRP genes in MYC_targets_V1. Each vertical dashed line represents a gene involved in the pathway. Each line is for one TRP gene. **b** The enrichment score (ES) distribution for the genes negatively co-expressed with TRP genes in MYC_targets_V1. **c** The enrichment score (ES) distribution for the genes positively co-expressed with TRP genes in MYC_targets_V2. **d** The enrichment score (ES) distribution for the genes negatively co-expressed with TRP genes in MYC_targets_V2.

In particular, we found that the majority of TRP genes were correlated with G2M checkpoint and MYC targets pathways (Figs. 6b, c and 7). The ability to sustain proliferation is a hallmark of cancer and the normal process of cell division occurs via the cell cycle^25^. Moreover, MYC family plays pivotal roles in the initiation and progression of human cancers^26,27^. Genes in TRPM family were more likely to be positively correlated with G2M checkpoint pathway, while genes in TRPC family were more likely to be negatively correlated pathway activity (Fig. 6b, c). These results were consistent with previous observations that TRPM2 silencing causes G2/M arrest and apoptosis in cancer^28^. Moreover, genes in TRPM family were more likely to be positively correlated with MYC targets pathway, while genes in TRPC family were more likely to be negatively correlated pathway activity (Fig. 7). The TRP-pathway correlations were validated in independent cancer datasets (Supplementary Fig. 11). Together, expression alterations of TRP genes are associated with patient survival and key oncogenic pathways.

### Potential therapeutic effects of TRP genes in cancer

**Fig. 8:**
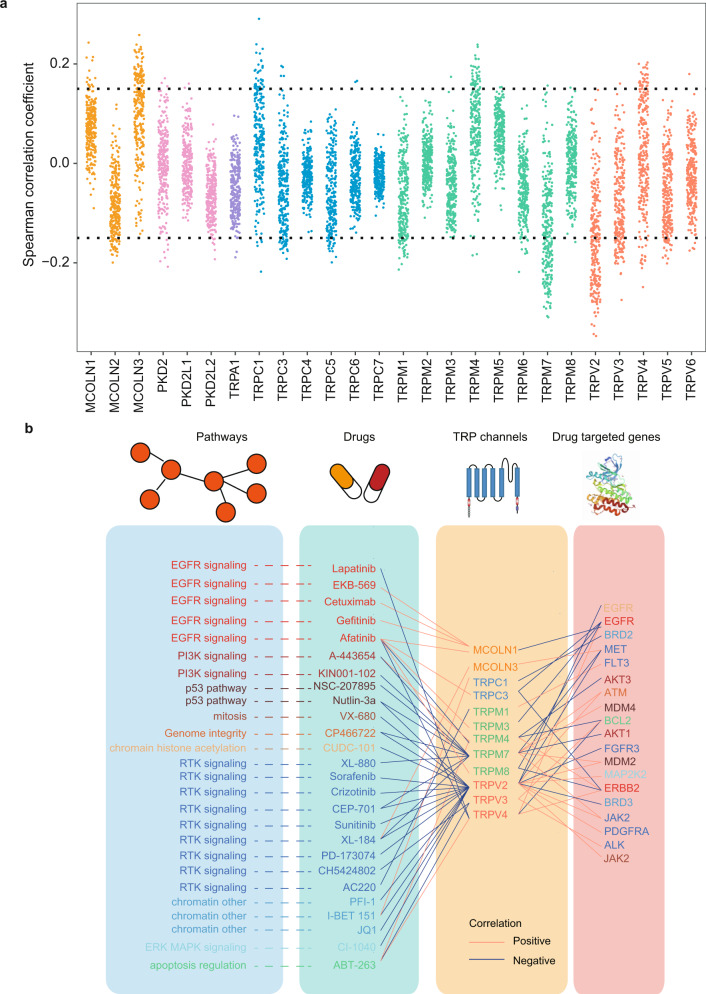
Potential drug targets of TPR genes in cancer. **a** The Spearman correlation coefficients between TRP gene expressions and drug IC50. **b** Correlation between TRP genes and known drugs that target the genes, which involved in number of cancer signaling pathways.

To further understand clinical implications of the TRP genes, we examined the correlations between transcriptional expressions of TRP genes and drug activities. We found that numbers of TRPs were correlated with drug activities across cell lines (Fig. 8a). In total, we identified 705 significant drug-TRP gene pairs involving 186 drugs and 23 TRP genes across cancer cell lines (Supplementary Table 5). Next, we focused on the TPRs that were also correlated with the expression of known drug targets. We observed 278 significant associations among 24 TRP genes and 86 known drug targets (Supplementary Fig. 12 and Supplementary Table 6).

In particular, we identified 12 TRP genes (e.g., TRPC1, TPC3, TRPM7, and TRPV2) correlated with known drug targets, such as EGFR, BRD2, AKT1, and ERBB2 (Fig. 8b). For example, recent studies have revealed that EGFR directly interacts with TRPM7 and play important roles in vascular homeostasis^29^. In addition, TRPM4 has been found to regulate AKT/GSK3-β activity and enhances β-catenin signaling and cell proliferation in cancer^30^. Moreover, we also investigated the potential effects of TRP genes on drug activities based on data from the Cancer Cell Line Encyclopedia (CCLE). We identified 20 significant drug-TRP gene pairs involving 7 drugs and 9 TRP genes across cancer cell lines (Supplementary Figs. 13, 14 and Supplementary Table 7). We also observed 20 significant associations among 9 TRP genes and 5 known drug targets (Supplementary Table 8). Therefore, the significant interactions between TRP genes and clinically actionable genes may affect the corresponding drug responses, suggesting that TRP genes play important roles in cancer therapy.

## Discussion

The emergence of evidence has revealed TRP channels as regulators in cancer growth and progression. However, the functional role of TRP channels across cancer types appears to be unclear, suggesting the need for comprehensive analyses. Here, we performed comprehensive analyses of the genetic alterations, transcriptome dysregulation, and potential clinical relevance of TRP genes across 33 cancer types. We found prevalent somatic mutations of TRP genes in cancer. In particular, the computational predicted deleterious mutations were likely to locate in the transmembrane region of TRP genes. Given the critical role of transmembrane regions of TRP genes^31^, cancer cells might selectively mutate the critical regions to perturb the signaling pathways.

Moreover, we observed that the genetic alterations of TRP genes were correlated with the transcriptome perturbations in cancer. The gene expression of TRP genes affected numerous cancer-related pathways. Cell proliferation is one of the major hallmarks of tumors. Moreover, we used the well-known proliferation marker MKI67 to reflect tumor proliferation across cancer samples. We found that multiple TRP genes were significantly associated with proliferation across cancers, suggesting their consistent roles in promoting or suppressing cell proliferation (Supplementary Fig. 15). To further confirm the functions of TRP genes in cell proliferation, we next analyzed the cell proliferation data from Project Achilles. We observed that the knockdown of several TRP genes decreased cell proliferation, such as PKD2, TRPM3, and TRPV5 (Supplementary Fig. 16). Taken together, our analysis suggested the important functions of TRP genes in cell proliferation.

In particular, we found that TRPM2 exhibited high expression across normal tissues, suggesting its housekeeping roles. This gene showed significantly higher expression across cancer types. Moreover, we also revealed several tissue or group-enriched genes (such as TRPM8, TRPA1, and TRPM3) in cancer. Tissue-specific gene expression is critical in understanding biological processes, physiological conditions, and disease^32^. We found that these tissue-specific or group-enriched TRP genes also exhibited perturbed expression in corresponding tissues. Collection and characterization of tissue-specific TRP genes at the molecular levels will be useful for understanding their oncogene’s roles, leading to the enhanced cancer therapy^33^.

Modulating the TRP channel activity in cancer provides an important way to regulate cellular function by altering of both membrane excitability and intracellular calcium levels^34^. Numbers of drugs or compounds that can modulate TRP channels, such as TRPV1, TRPV3, TRPV4, and TRPA1 have all entered clinical trials. We revealed numbers of candidate drugs, of which activities were correlated with the expression of TRP genes, suggesting promising targets for drug discovery. Although gene expression is usually considered as an important indication of gene function, it doesn’t mean that the gene expression change is functionally related to drug response. Next, we calculated the correlations between gene essentiality and drug IC50 based on the Depmap data^35^. We found that there were 5 significant correlations among 4 TRP genes and 4 drugs. These results indicated the therapeutical effects of TRP genes provided candidates. However, we only revealed the correlations and several computational methods (such as My Personal Mutanome and GPSnet^34,35^) can be applied to the TRP gene panels for investigating the genotype-phenotype relationship and drug repurposing. Future cell line or animal models are needed to verify these candidate drugs before clinical administration.

In conclusion, genome-wide analysis of the somatic mutations, CNV, and transcriptome supported the role of TPR channels in tumorigenesis. Our study provided a comprehensive genetic and pharmacogenomics landscape of TRP channels across cancer types, which will shed light on the future development of therapeutic targets.

## Methods

### Collection of TRP channels

Transient-receptor potential channels (TRP channels) are a group of ion channels. We collected the TRP genes from HGNC^36^ (https://www.genenames.org/data/genegroup/#!/group/249). There are 28 TRP channels that share some structural similarity with each other. Genes were classified into different sub-families based on the annotation from HGNC.

### Evolution tree of TRP channels

We obtained the sequences of 28 TRP genes from GeneBank database^37^. The evolutionary history of TRP channels was inferred using the Neighbor-Joining method^38^. The optimal tree with the sum of branch length = 13.52 is shown. The evolutionary distances were computed using the Maximum Composite Likelihood method^39^ and are in the units of the number of base substitutions per site. The analysis involved 28 nucleotide sequences. Codon positions included were 1st + 2nd + 3rd + Noncoding. All positions containing gaps and missing data were eliminated. There were a total of 312 positions in the final dataset. Evolutionary analyses were conducted in MEGA7^40^.

### Somatic mutations and copy number variation of cancer patients

Genome-wide somatic mutations were downloaded from UCSC Xnea^41^ and the ‘MC3’ mutation calls file was downloaded (https://tcga-pancan-atlas-hub.s3.us-east-1.amazonaws.com/download/mc3.v0.2.8.PUBLIC.xena.gz). Somatic mutations over 10,000 tumors across 33 different cancer types were obtained from The Cancer Genome Atlas (TCGA)^42^. Seven mutation-calling algorithms with scoring and artifact filtering were performed to get the mutations^43^. The mutation frequency of gene or mutation was calculated as the proportion of samples with mutation in a specific cancer. Moreover, we obtained the somatic mutations across 31 cancer types from International Cancer Genome Consortium (ICGC) (https://dcc.icgc.org/)^44^.

The gene-level CNV data for 33 cancer types were also downloaded from UCSC Xnea. The CNV profiles were derived from GISTIC2^45^. GISTIC2 further thresholded the estimated CNV values to −2, −1, 0, 1, and 2, representing homozygous deletion, single copy deletion, diploid normal copy, low-level copy number amplification, or high-level copy number amplification^45^. The CNV amplification and loss frequency of gene was calculated as the proportion of samples with CNV amplification or loss in a specific cancer.

### Deleterious and damaging mutations

The transmembrane regions of TRP genes were downloaded from UniProt^46^. We calculated the mutation density of transmembrane regions and other regions. For the transmembrane regions (TRs) in TRP genes, we assume that the observed number of mutations for TRs follows a binomial distribution^47^. The binomial is $(N,p_{ri})$, in which *N* is the total number of mutations observed in one gene and $p_{ri}$ is the expected mutation rate for the TRs. The null hypothesis is that the region was not recurrently mutated. We defined $L_{TR}$ to represent the length of the TRs and $L_g$ is the length of gene. For TRs in a gene, we calculated the *P*-value, which is the probability of observing ≥*k* mutations in the TRs out of *N* total mutations observed in this gene:

1 $$P(X \ge k) = 1 - P(X \,<\, k) = 1 - \mathop {\sum}\limits_{x = 0}^{k - 1} {\left( {\frac{N}{x}} \right)} \;p_{ri}^x(1 - p_{ri})^{N - x}$$

where $p_{ri} = \frac{{L_{TR}}}{{L_g}}$. In addition, we calculated the enrichment ratio for TRs as follows:

2 $$E = \frac{k}{{N \ast L_{TR}/L_g}}$$

The mutation density of TRs and other regions were compared with Wilcoxon’s rank-sum test. TRP genes with *E* > 1.3 and *p*-value were identified as TRPs enriching mutations in TRs. We found that PKD2L1, TRPM1, TRPM2, and TRPM3 were with mutations enriching in TRs.

Moreover, the mutation effects on protein structure were assessed by SIFT^48^ and PolyPhen-2^49^. The proportion of deleterious mutations in TRs and other regions was compared with Fisher’s exact tests. CADD scores of mutations were calculated^50^ and we used Wilcoxon’s rank-sum test for comparing the differences between TRs and other regions.

### Differential expression analysis of TRP channels

The gene expressions across 33 cancer types were downloaded by the R package TCGAbiolinks^51^. Gene expression was measured by Fragments Per Kilobase of exon model per Million mapped fragments (FPKM). The expressions of genes were log-transformed. Differential expression analysis was performed in 18 cancer types with >= normal samples. Wilcoxon’s rank-sum test was used to evaluate the differences in expression between normal and cancer samples. Genes with log2(fold-changes) >1 and *p*-adjusted <0.05 were identified as differentially expressed genes. Moreover, the expression correlations between TRP genes were calculated by ‘cor’ function in R program.

Moreover, we downloaded the gene expression profiles of 11 cancer types from ArrayExpress (E-MTAB-6690, pancreatic cancer; E-MTAB-6691, ovarian cancer; E-MTAB-6692, renal cancer; E-MTAB-6693, gastric cancer; E-MTAB-6694, prostate cancer; E-MTAB-6695, liver cancer; E-MTAB-6696, bladder cancer; E-MTAB-6697, melanoma cancer; E-MTAB-6698, colorectal cancer; E-MTAB-6699, lung cancer; and E-MTAB-6703, breast cancer). All the gene expressions were RMA normalized, merged, and batch effected via Combat method. Another six gene expression profiles and corresponding clinical information were downloaded from Gene Expression Omnibus under the accession numbers GSE57495 (pancreatic cancer), GSE42127 (non-small-cell lung cancer), GSE30219 (metastatic-prone tumors), GSE28735 (pancreatic ductal adenocarcinoma), GSE23554 (Ovarian Cancer), and GSE17536 (Colon Cancer).

### Tissue-specific expression of TRP genes

Tissue-specific expression of TRP genes was analyzed based on human protein atlas^52,53^. Genes were classified into different groups based on their expression across normal tissues^21^.

### Clinical features of patients

The clinical features of patients, including sex, stage, ethnicity, grade, weight, and survival time were obtained from TCGA project. The tumor mutation burden (TMB) is calculated as the total number of nonsynonymous mutations in each patient. The hypoxia scores were obtained from cBioPortal.

### Functional pathways analysis

To identify the potential functional pathways of TRP genes, we calculated the expression correlation of all other genes with each TRP gene. All protein-coding genes were ranked based on the Spearman correlation coefficients. The ranked gene lists were subjected into gene set enrichment analysis (GSEA)^54,55^. Cancer hallmark pathways from MSigDB were used in our analysis^56^.

### Clinical relevance of TRP genes

Clinical data of tumor patients across 33 cancer types were downloaded from UCSC xena database. We used the ‘survfit’ function in survival R package to calculate the Hazard Ratio (HR) of each TRG gene. The overall survival times of patients were used. If TRP genes were with 0 expressions in more than 50% tumor samples, these TRP genes were not analyzed. Patients were classified into two groups based on the ‘surv_cutpoint’ function. Genes with HR > 1 and *p* < 0.05 were risky factors and genes with HR < 1 and *p* < 0.05 were protective factors.

### Drug activities analysis

The IC50 data of drugs across cell lines were downloaded from Genomics of Drug Sensitivity in Cancer (GDSC)^57^. Moreover, we also downloaded the gene expression of cell lines from GDSC. Only 26 TRP genes were included in this data. We next excluded the drugs with ‘NA’ for IC50 in more than 30% cell lines. The missing values of IC50 were imputed by KNN method (k = 5, rowmax = 0.5, colmax = 0.8, maxp = 1500, rng.seed = 362436069). The Spearman correlation coefficients (SCC) between TRP expression and IC50 were calculated for TRP-drug pairs. The pairs with |SCC| > 0.15 and *p-adjusted* < 0.05 were identified.

Moreover, we also collected 123 clinical actionable genes from one recent study^58^. There were 117 clinical actionable genes were with expression in cell lines. The SCCs between TRP genes and clinical actionable genes were calculated and the gene pairs with |SCC| > 0.2 and adjusted *p* < 0.05 were identified. In the river plot, we used the |SCC| > 0.15 for visualization. The drug-target and corresponding pathways information for targets were also obtained from GDSC.

We also downloaded the drug IC50 and gene expression across cell lines from the Cancer Cell Line Encyclopedia (CCLE)^59^. We performed similar analyses as GDSC and obtained the drug-TRP-target correlation across cell lines.

### Gene essentiality and tumor proliferation analysis

The gene essentiality data were downloaded from Depmap (https://depmap.org/portal/download)^35^. We estimated the gene dependency across cell lines based on the Bayesian inference^60^. We then assessed the association between individual TRP expressions and gene essentiality scores by Spearman’s correlation and considered |Rs| > 0.2 and adjusted *p*-values < 0.05 to indicate significance.

We used the well-known proliferation marker MKI67 to reflect tumor proliferation across TCGA samples. The correlations of TRP expressions and MKI67 expression were assessed by Spearman’s correlation. We also applied this method in cancer cell lines similar to one previous study.

### Reporting summary

Further information on research design is available in the Nature Research Reporting Summary linked to this article.

## Supplementary information

Supplementary figures

Supplementary Table 1

Supplementary Table 2

Supplementary Table 3

Supplementary Table 4

Supplementary Table 5

Supplementary Table 6

Supplementary Table 7

Supplementary Table 8

Reporting Summary

## Data Availability

Data and download URLs involved in this study had been described in detail in the Methods section. All results generated in this study can be obtained by contacting the corresponding authors on reasonable request.

## Code availability

All the bioinformatics analyses involved in this study were based on R program, and the used R packages were described in detail in the Methods section. The codes can be downloaded from https://github.com/ComputationalEpigeneticsLab/Transient-Receptor-Potential-Channels-Analysis.
