# Supplementary material for: Pan-cancer analyses reveal the genetic and pharmacogenomic landscape of transient receptor potential channels
Source: NPJ Genom Med. 2022 May 25;7:32. doi: 10.1038/s41525-022-00304-1 (PMC9132893; doi:10.1038/s41525-022-00304-1)
Supplement: Supplementary file 1 — Supplementary figures [file 41525_2022_304_MOESM1_ESM.docx]

**Supplementary figures**

**
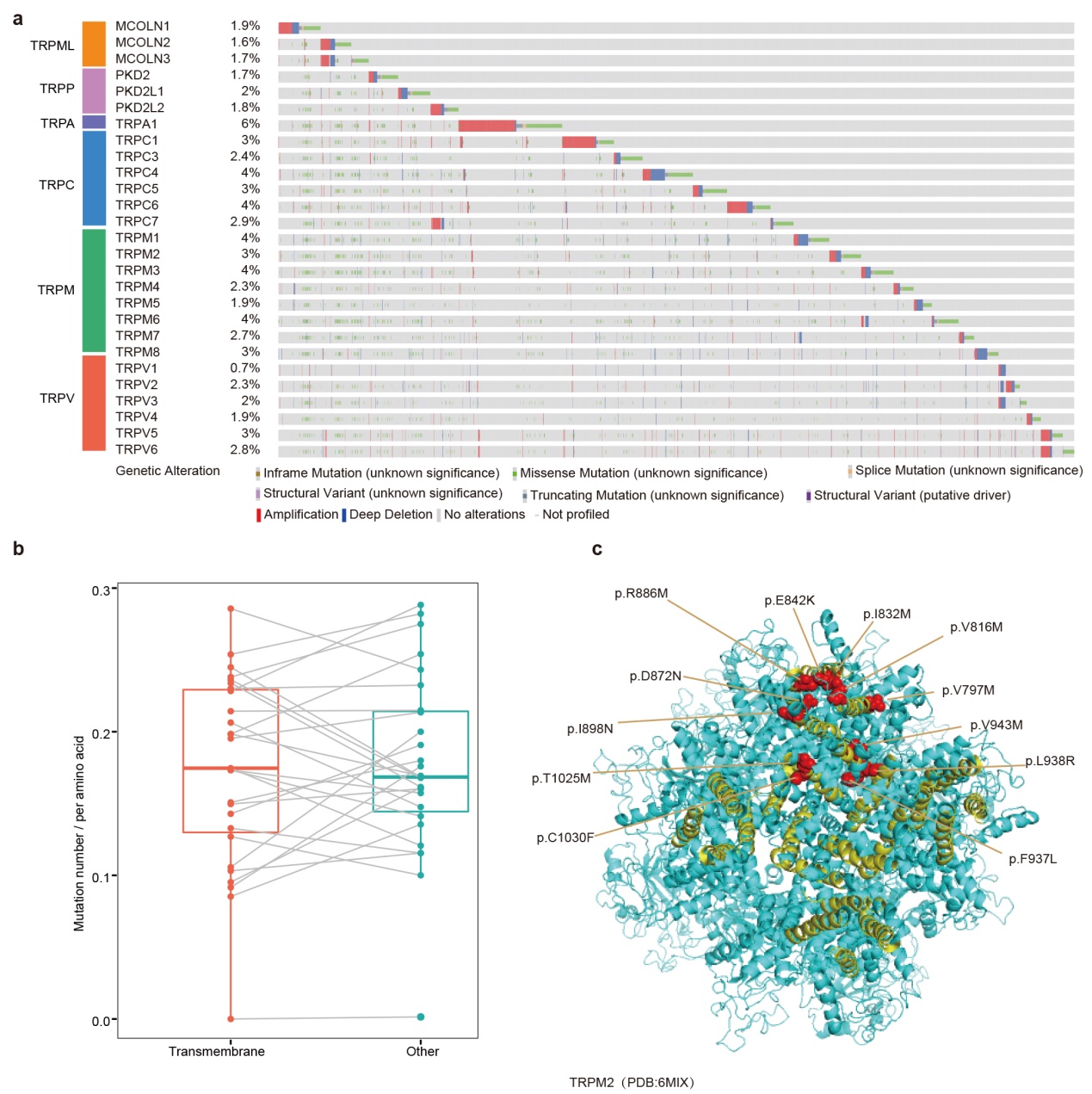
**

**Supplementary Fig. 1. Genetic alterations of TRPs across cancer types. a** Waterfall plot showing the mutations and CNV alterations of TRPs across cancer types. Data of TCGA pan-cancer project were downloaded from cBioportal. **b** The mutation density of transmembrane regions and other regions. **c** PDB structure of TRPM2. Deleterious or damaging mutations were marked with red balls.


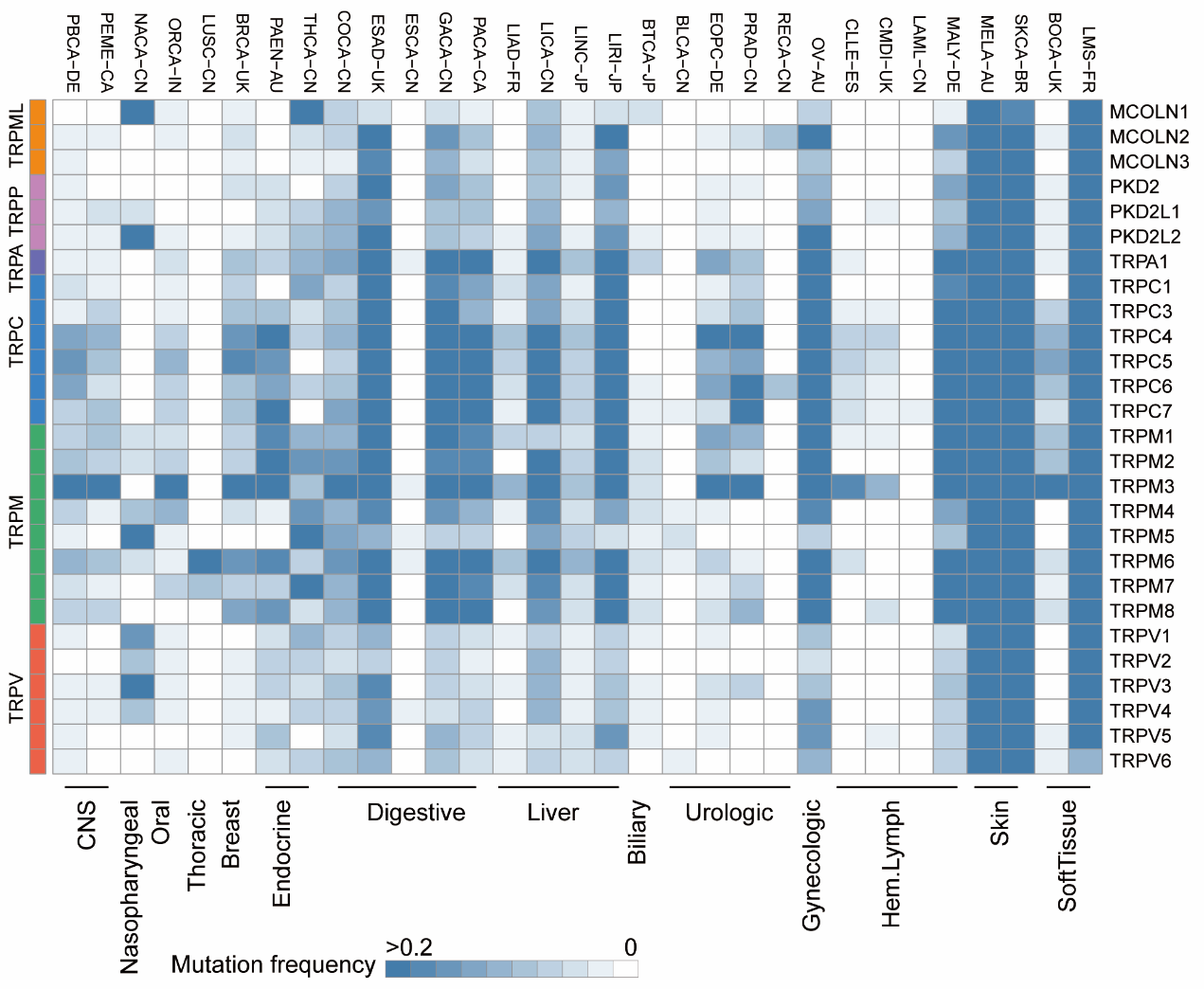


**Supplementary Fig. 2.** **Mutation frequency profile of TRPs across cancer types.** The heat map of mutation frequency of TRPs across tissues. Data were downloaded from International Cancer Genome Consortium.


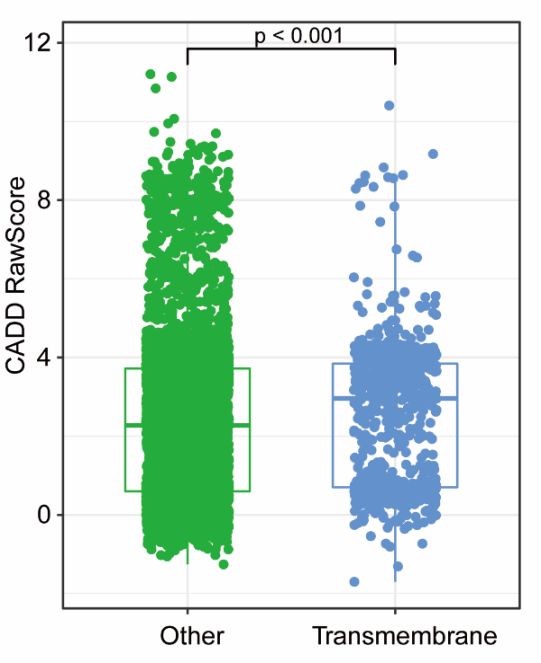


**Supplementary Fig. 3. CAD scores of the mutations in transmembrane regions and other regions of TPR genes**. P-value for Wilcoxon’s rank sum test.

**
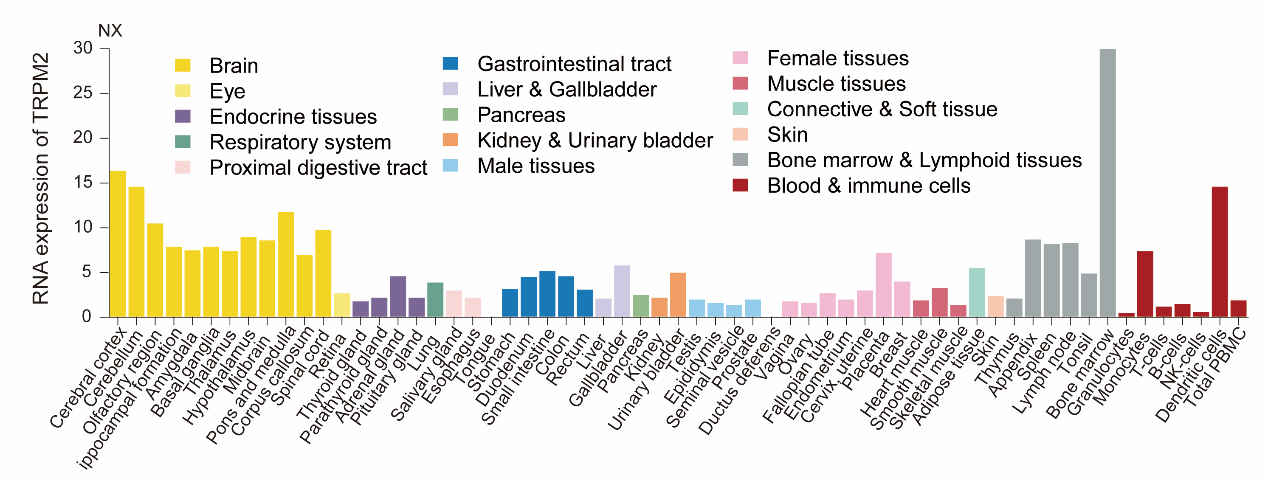
**

**Supplementary Fig. 4.** **mRNA expression of TRPM2 across tissues.** RNA expression of TRPM2 across tissues. Data were downloaded from human protein atlas.


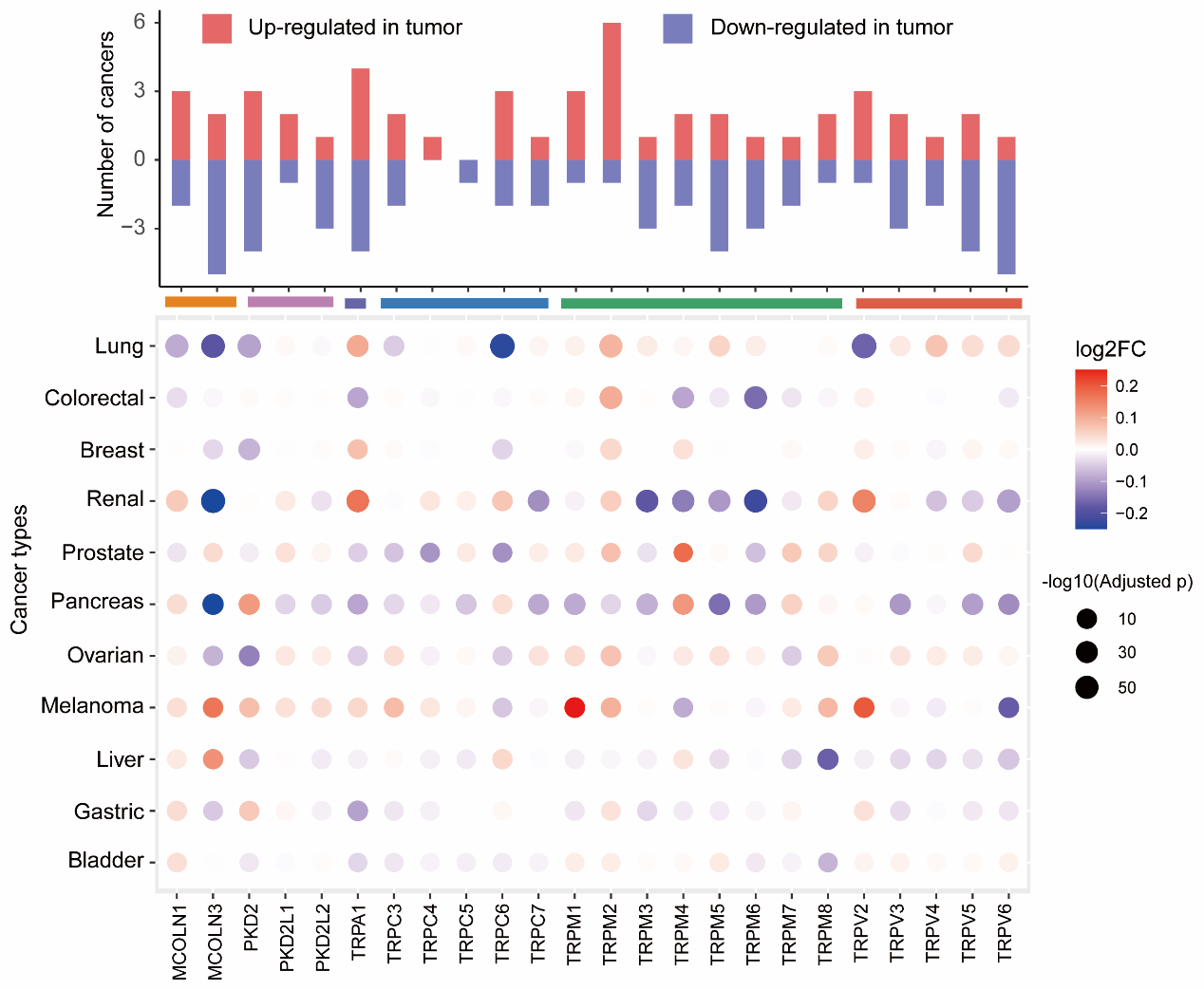


**Supplementary Fig. 5. Transcriptome dysregulation of TPR genes in cancer.** Bar plots showing the number of cancer types that each TRP gene exhibited up-regulation or down-regulation. The heat map at the bottom showing the fold-changes of TPRs in comparison between tumor and normal.


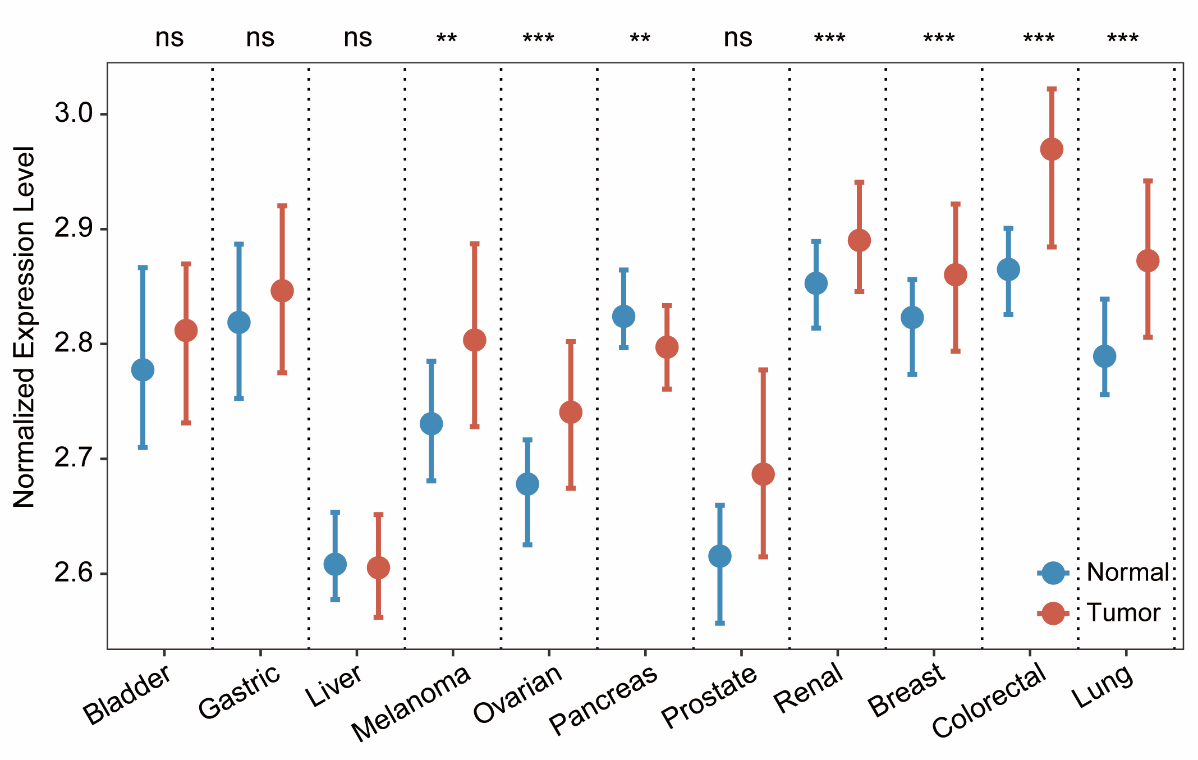


**Supplementary Fig. 6. mRNA expression of TRPM2 protein between tumor and normal tissues.** RNA expression of TRPM2 across tissues. Data were downloaded from ArrayExpress.


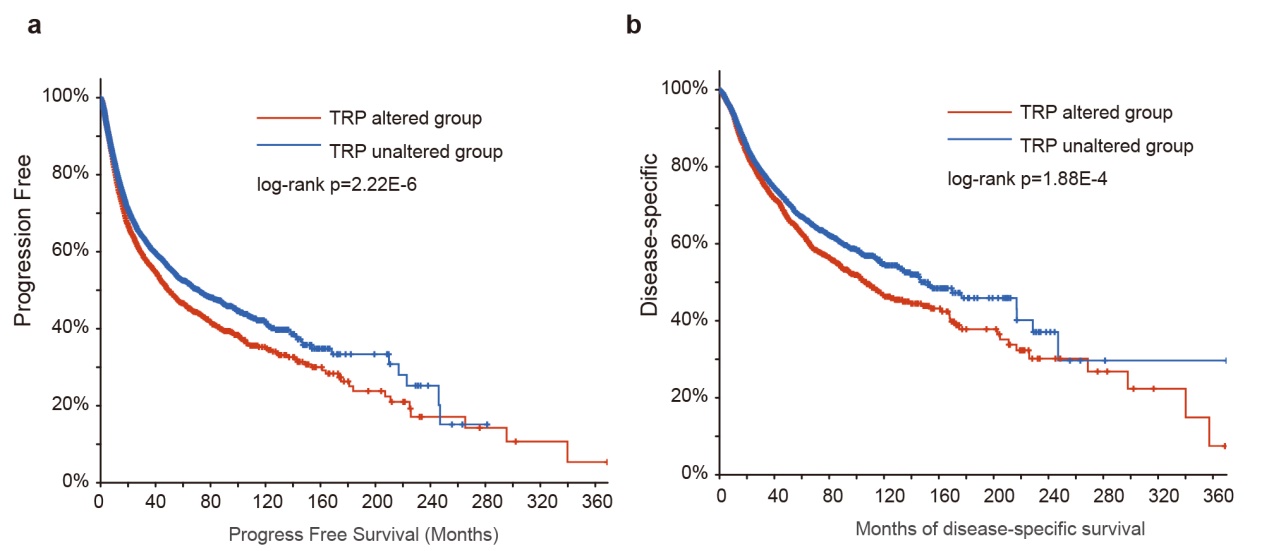


**Supplementary Fig. 7. Kaplan-Meier survival plot of patients grouped by with or without TRP genetic alterations.** Left for progression free survival and right for disease specific survival.


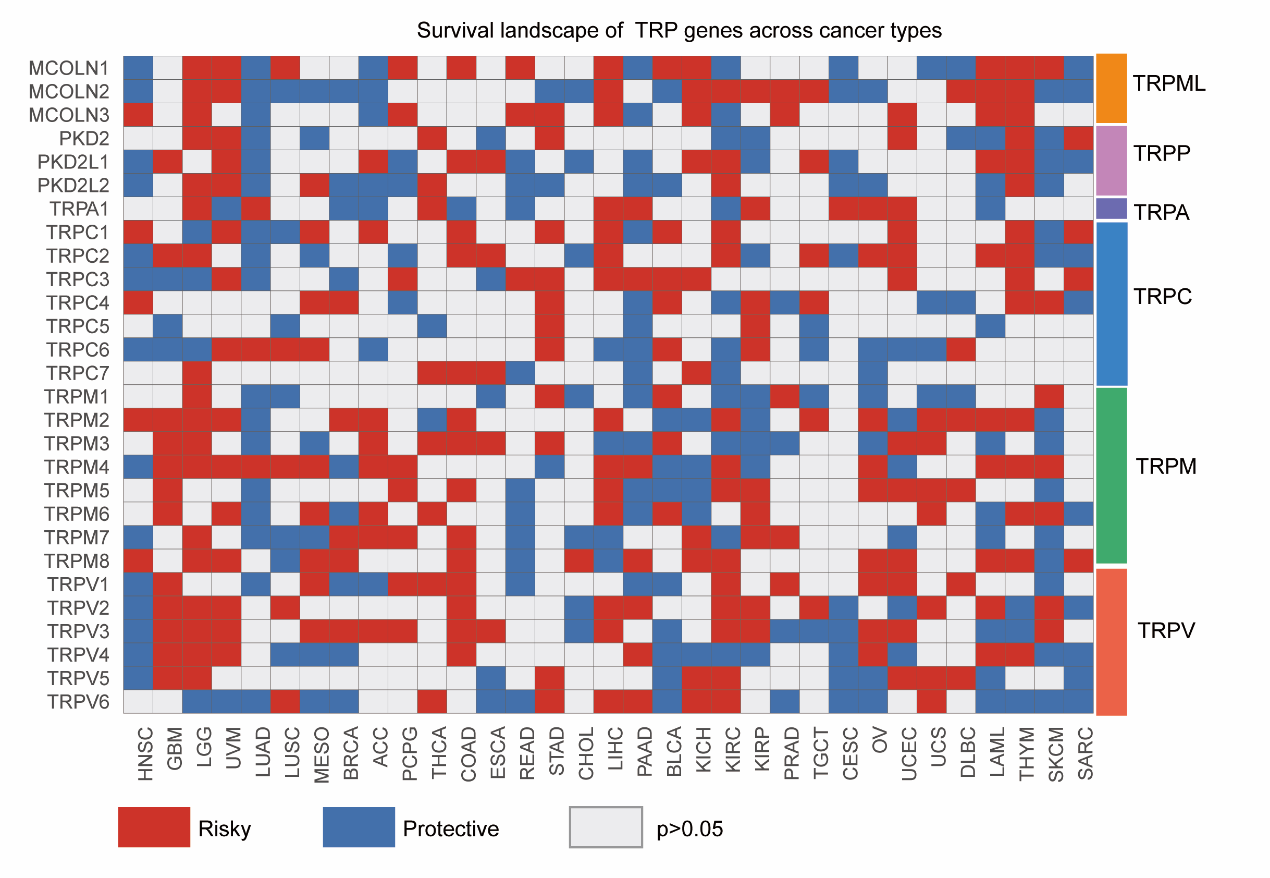


**Supplementary Fig. 8. Clinical relevance landscape of TRPs across cancer types.** Red indicates the high expression of TRP gene is associated with poor survival. Blue indicates the high expression of TRP gene is associated with better survival.


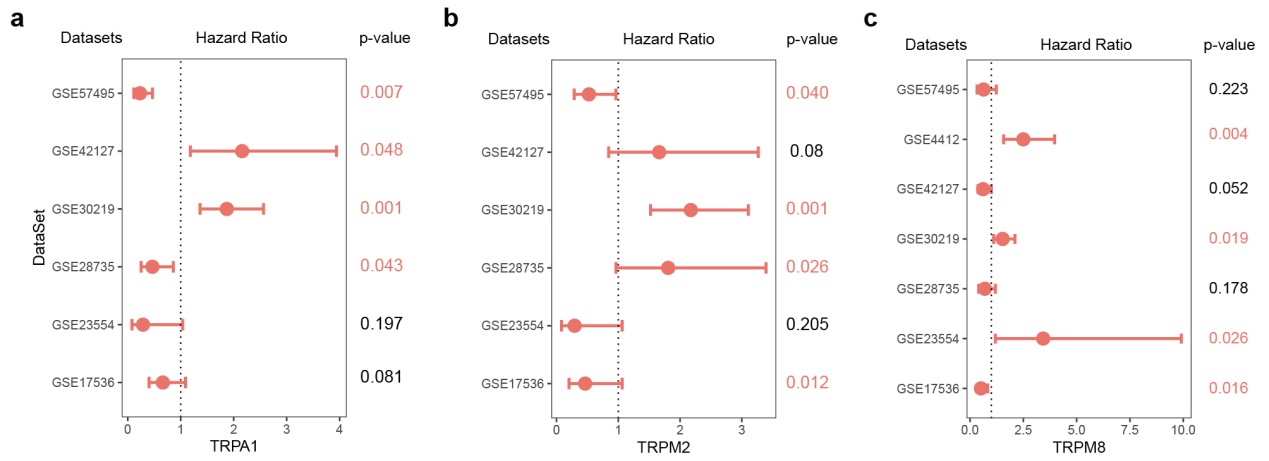


**Supplementary Fig. 9. The distribution of hazard ratios across different cancer types.** **a** The distribution of hazard ratios based on TRPA1 expression across different cancer types. **b** The distribution of hazard ratios based on TRPM2 expression across different cancer types. **c** The distribution of hazard ratios based on TRPM8 expression across different cancer types.


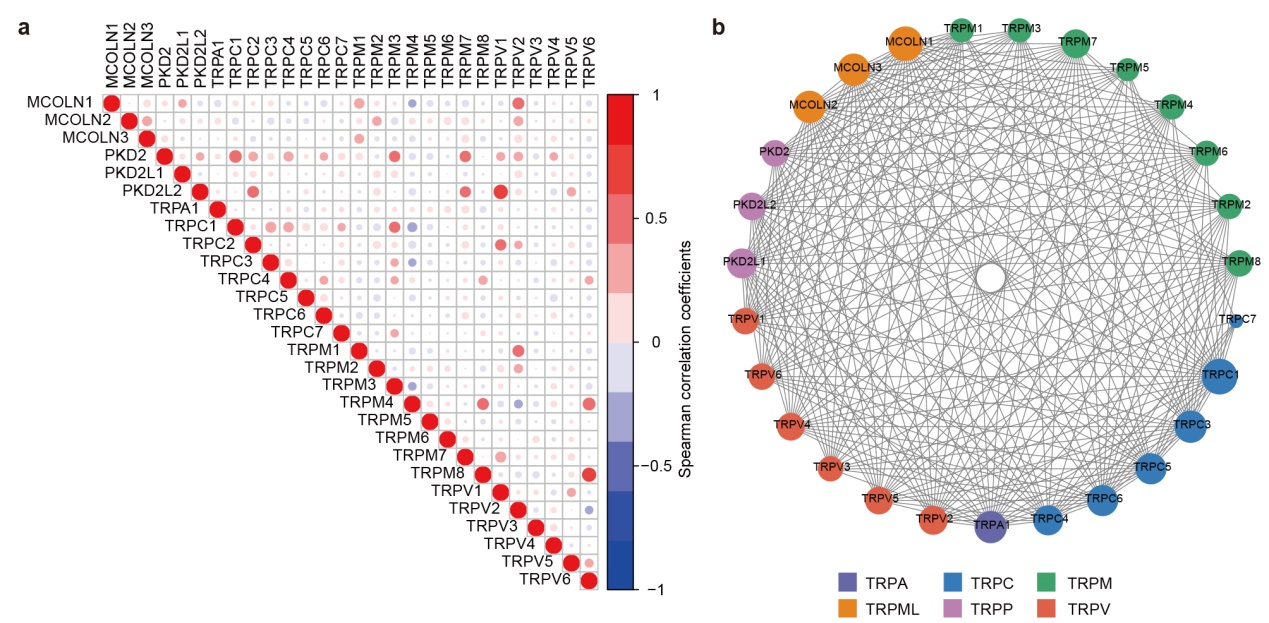


**Supplementary Fig. 10. Co-expression and protein interactions of TRPs.** **a** Heat map showing the co-expression among TRP genes across cancer types. **b** Protein-protein interactions among TRPs. Data was downloaded from STRING database.


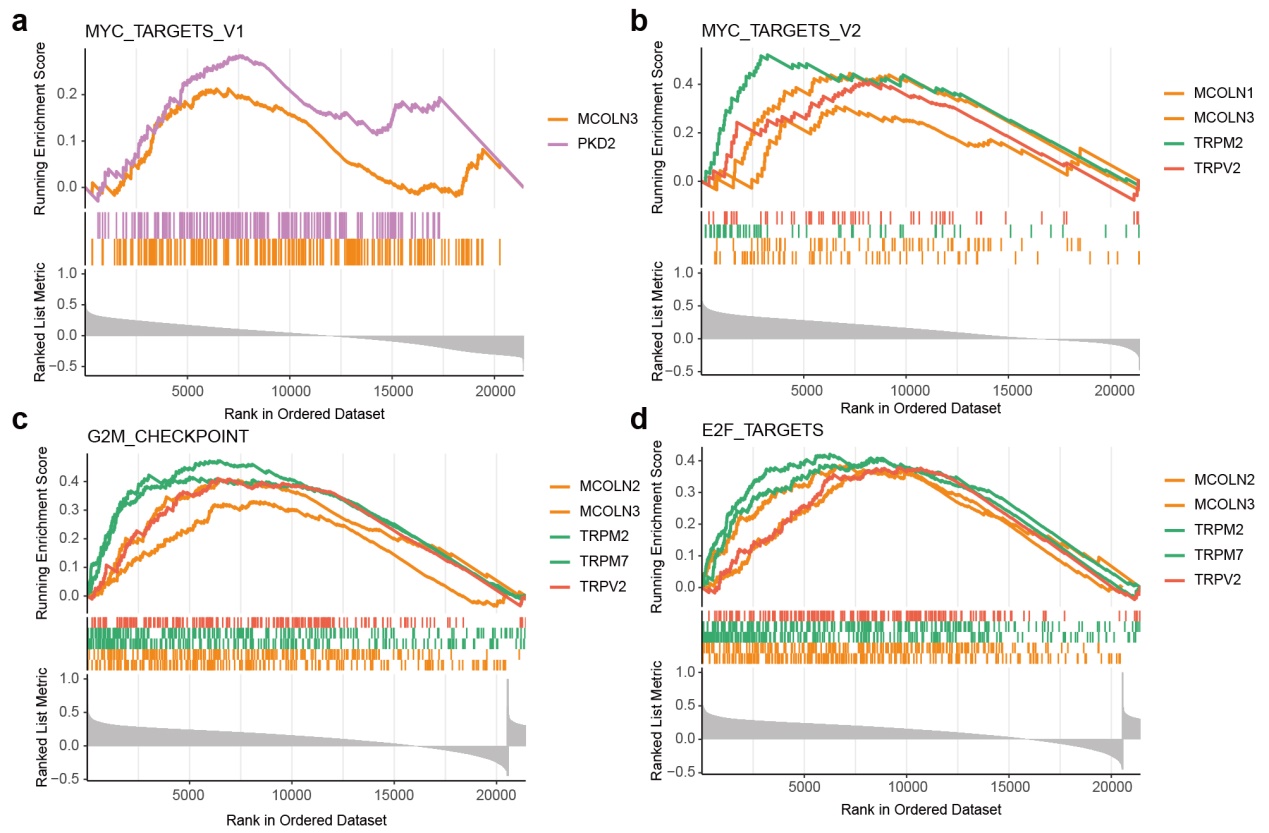


**Supplementary Fig. 11. Genes co-expressed with TRPs are enriched in cancer pathways.** **a** The enrichment score (ES) distribution for the genes positively co-expressed with TRP genes in MYC_TARGETS_V1. Each vertical dashed line represents a gene involved in the pathway. Each line is for one TRP gene. **b** The enrichment score (ES) distribution for the genes positively co-expressed with TRP genes in MYC_ TARGETS_V2. **c** The enrichment score (ES) distribution for the genes positively co-expressed with TRP genes in G2M_CHECKPOINT. **d** The enrichment score (ES) distribution for the genes positively co-expressed with TRP genes in E2F_TARGETS.


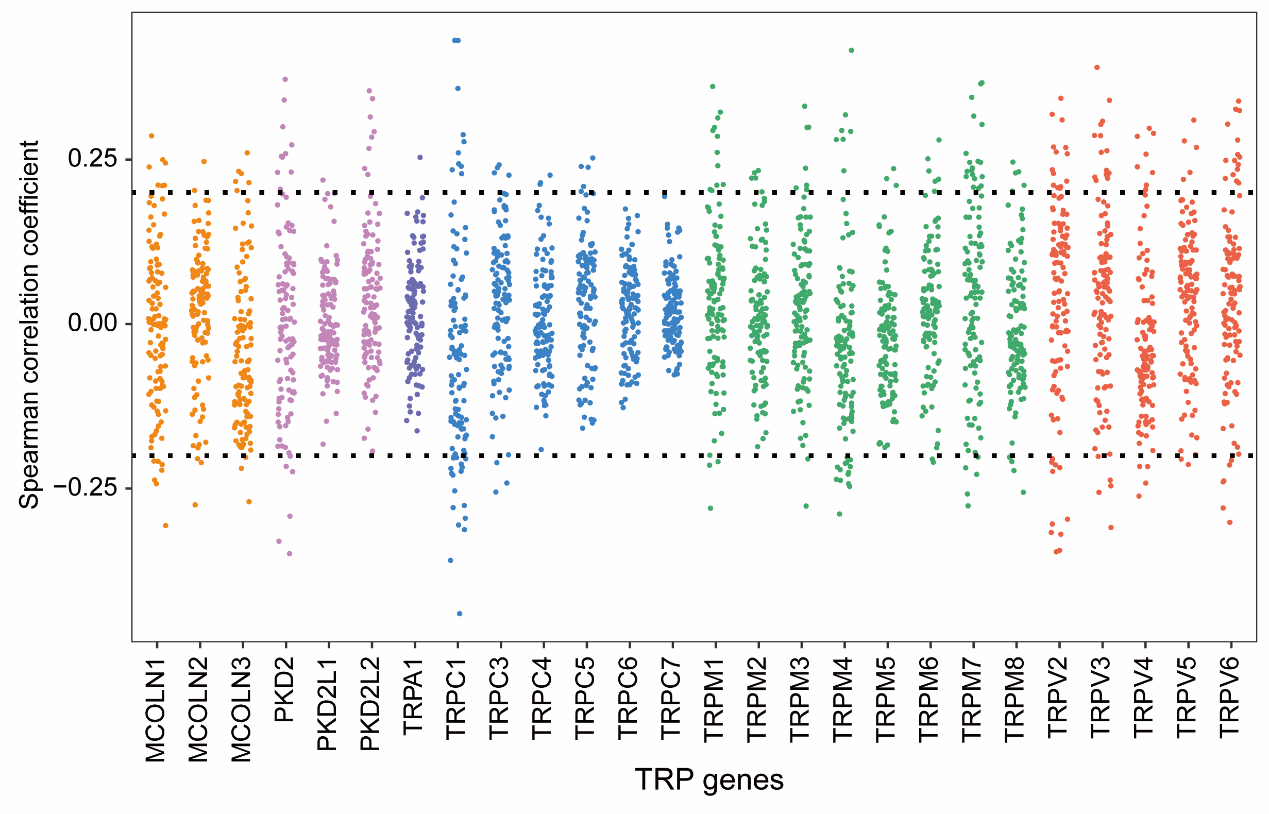


**Supplementary Fig. 12. Expression correlation between drug target and TRP genes across cancer cell lines.** The spearman correlation coefficients between TRP gene expressions and drug IC50.


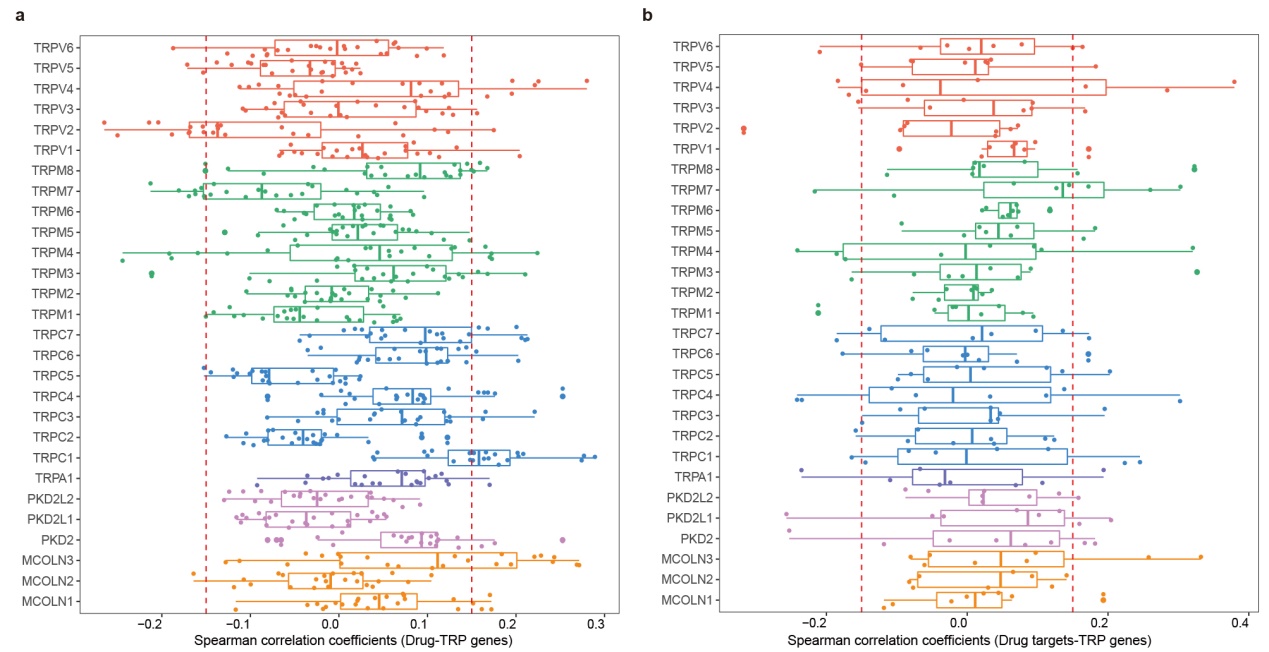


**Supplementary Fig. 13. Correlation between drug IC50, target gene expression and TRP gene expression across cancer cell lines in CCLE. a** Correlation between drug IC50 and TRP gene expression. **B,** Correlation between expressions of TRP gene and drug targets.


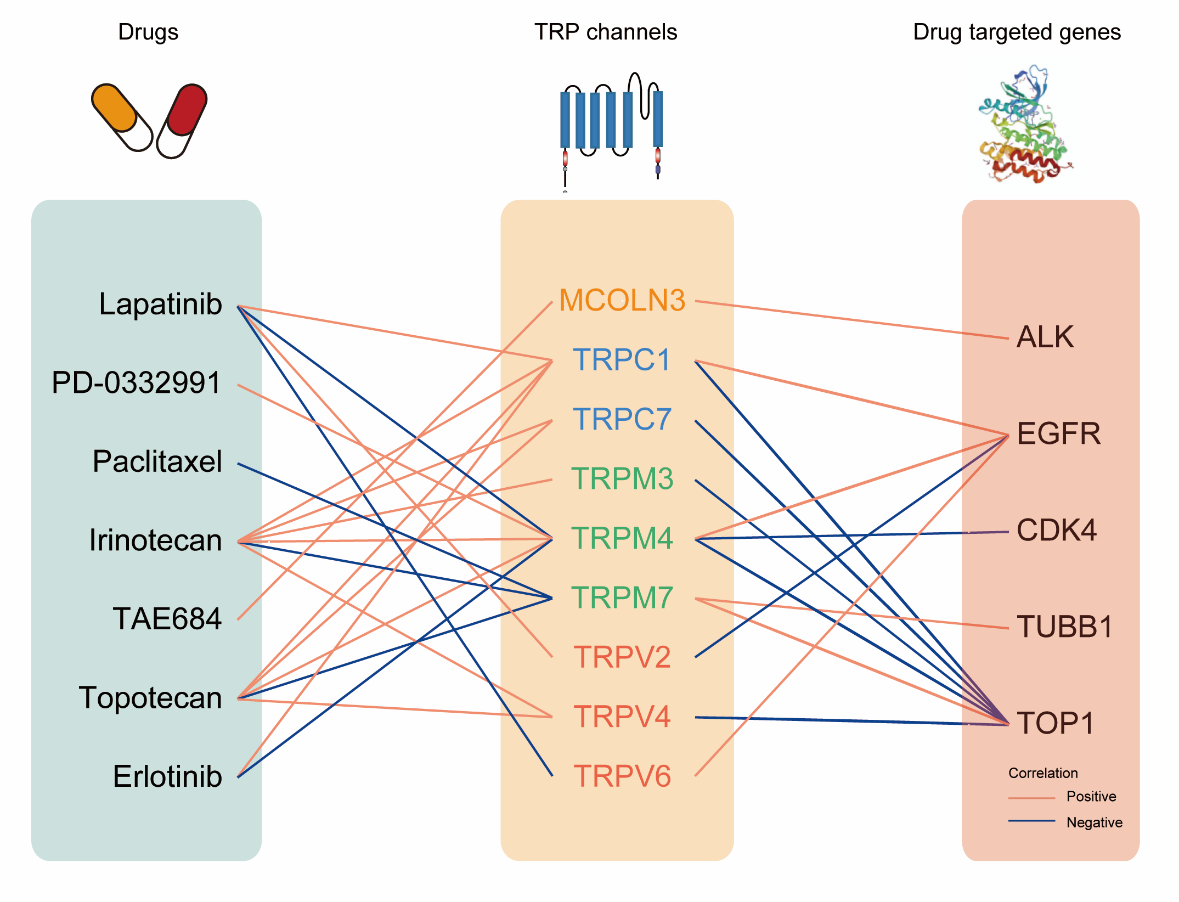


**Supplementary Fig. 14. Correlation between drugs-TRP genes and drug targeted genes in CCLE.** River plot shows the correlation between TRP genes and known drugs that target the genes.


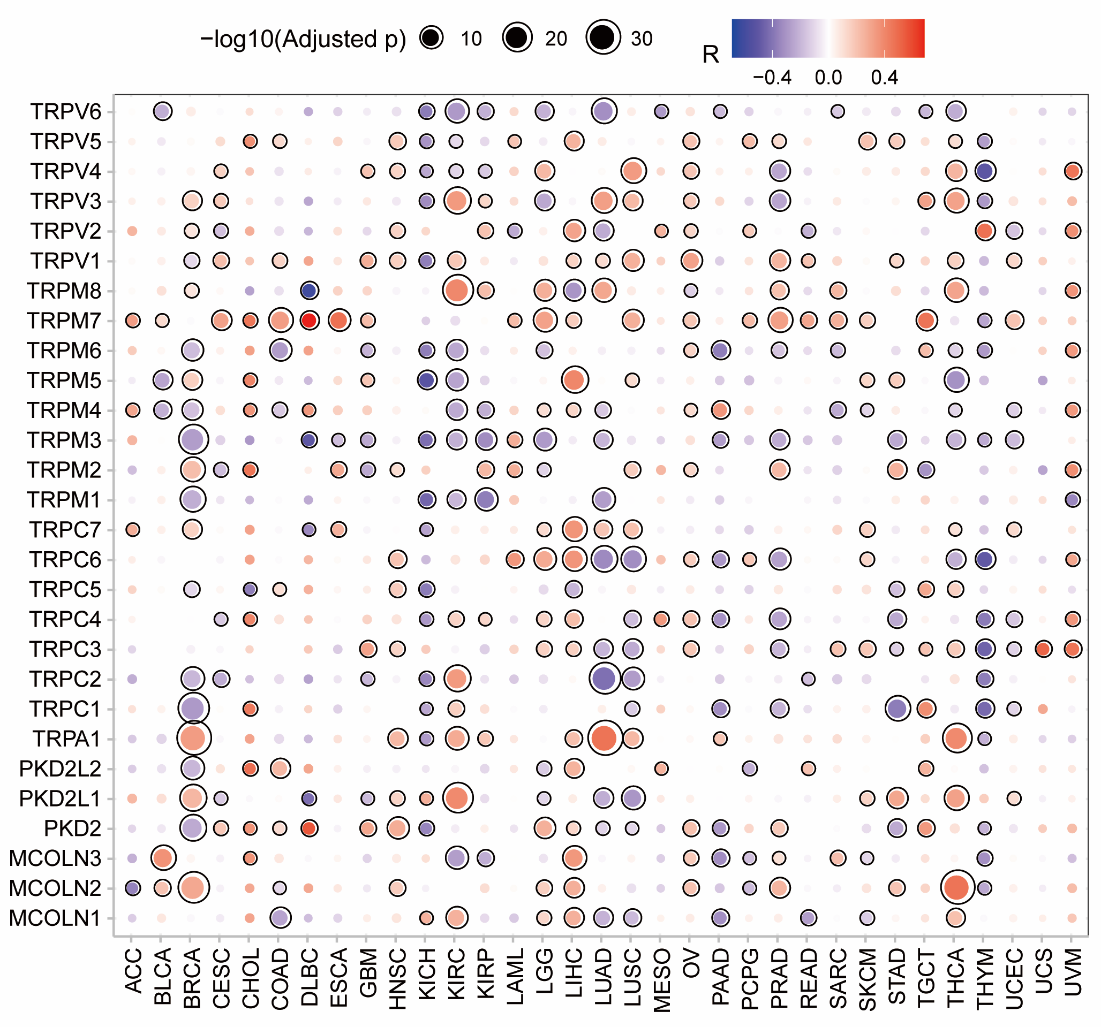


**Supplementary Fig. 15. Dual functional effects of TRPs in cell proliferation.** Correlation between TRPs and proliferation marker (MKI67) across cancer types. Red and blue colored circles indicate positive and negative correlations.


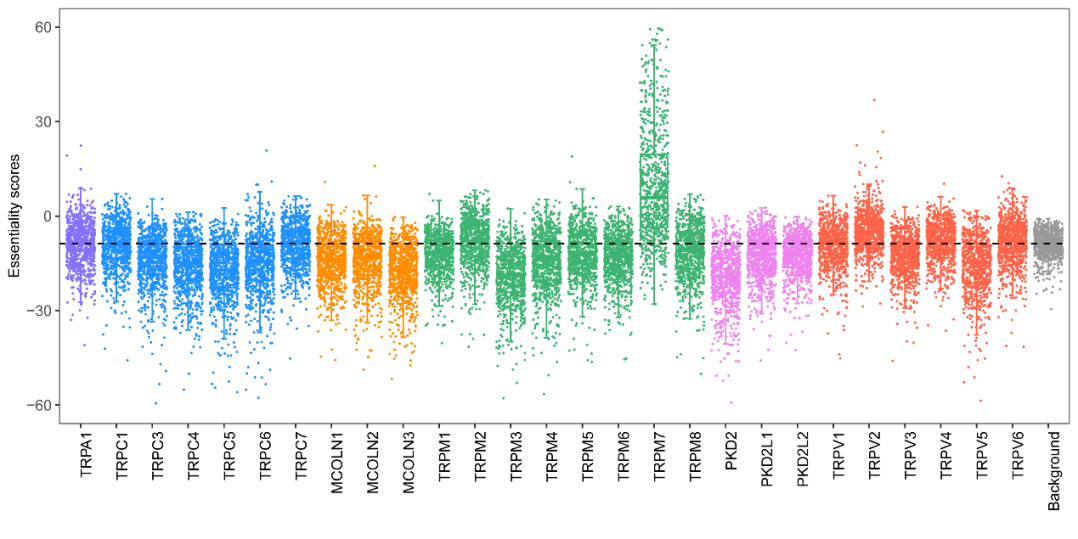


**Supplementary Fig. 16. Gene essentiality analysis.** Significant decrease in gene essentiality in cancer cell lines by knocking out individual TRP, data from Project Achilles.
